# Human Biomonitoring of Selected Hazardous Compounds in Portugal: Part I—Lessons Learned on Polycyclic Aromatic Hydrocarbons, Metals, Metalloids, and Pesticides †

**DOI:** 10.3390/molecules27010242

**Published:** 2021-12-31

**Authors:** Angelina Pena, Sofia Duarte, André M. P. T. Pereira, Liliana J. G. Silva, Célia S. M. Laranjeiro, Marta Oliveira, Celeste Lino, Simone Morais

**Affiliations:** 1 LAQV, REQUIMTE, Laboratory of Bromatology and Pharmacognosy, Faculty of Pharmacy, University of Coimbra, Polo III, Azinhaga de Sta Comba, 3000-548 Coimbra, Portugal; apena@ci.uc.pt (A.P.); andrepereira@ff.uc.pt (A.M.P.T.P.); ljgsilva@ff.uc.pt (L.J.G.S.); celialaranjeiro@gmail.com (C.S.M.L.); clino@ff.uc.pt (C.L.); 2 Centro de Investigação Vasco da Gama-Departamento de Ciências Veterinárias, Escola Universitária Vasco da Gama, Av. José R. Sousa Fernandes, Campus Universitário-Bloco B, 3020-210 Coimbra, Portugal; 3 LAQV/REQUIMTE, Instituto Superior de Engenharia do Porto, Instituto Politécnico do Porto, Rua Dr. António Bernardino de Almeida 431, 4249-015 Porto, Portugal; Marta.Oliveira@graq.isep.ipp.pt (M.O.); sbm@isep.ipp.pt (S.M.)

**Keywords:** biomarkers of exposure, metals, metalloids, pesticides, polycyclic aromatic hydrocarbons (PAHs), health risks

## Abstract

Human biomonitoring (HBM) data provide information on total exposure regardless of the route and sources of exposure. HBM studies have been applied to quantify human exposure to contaminants and environmental/occupational pollutants by determining the parent compounds, their metabolites or even their reaction products in biological matrices. HBM studies performed among the Portuguese population are disperse and limited. To overcome this knowledge gap, this review gathers, for the first time, the published Portuguese HBM information concerning polycyclic aromatic hydrocarbons (PAHs), metals, metalloids, and pesticides concentrations detected in the urine, serum, milk, hair, and nails of different groups of the Portuguese population. This integrative insight of available HBM data allows the analysis of the main determinants and patterns of exposure of the Portuguese population to these selected hazardous compounds, as well as assessment of the potential health risks. Identification of the main difficulties and challenges of HBM through analysis of the enrolled studies was also an aim. Ultimately, this study aimed to support national and European policies promoting human health and summarizes the most important outcomes and lessons learned through the HBM studies carried out in Portugal.

## 1. Introduction

In everyday life, humans are exposed to a broad range of hazardous substances and their mixtures, present in air, soil, water, and food. It is of utmost importance to ensure scientific evidence in order to allow early protection of human health, since some of these chemicals cause deleterious effects, and prolonged human exposure, even at low doses, can be linked with chronic diseases and cancer [1]. Different approaches can be followed, namely the assessment of environmental/occupational levels of hazardous pollutants and food contaminants, and/or the determination of cumulative chemical burden through human biomonitoring (HBM) actions [2]. HBM represents an adequate tool to assess human exposure to hazardous substances and/or their associated health risks through the measurement of chemicals, their metabolites or reaction products in biological matrices (e.g., blood, urine, breast milk, saliva, etc.) [3]. HBM studies allow the determination of total exposure to mixtures of contaminants/pollutants that are causing growing concern in human health risk assessment regardless of the route of exposure (inhalation, ingestion, or dermal uptake) and taking into account personal characteristics and individual lifestyles [4,5]. HBM can help find: (1) new emerging chemical exposures, as well as new tendencies and variations in such exposure; (2) populations or groups more vulnerable or with higher exposures; (3) the patterns of exposure not only among the general population but also among specific population groups. The use of HBM studies can help in clarifying the association between environmental/occupational exposure and personal internal exposure and early health risks; however, the sources and routes of exposure cannot be identified, and neither can any causal correlation be established. When performed over time, HBM studies allow the assessment of exposure trends, and comparison of the data obtained with the available reference guidelines and/or with the values obtained for control groups will allow, if necessary, corrective actions [6]. Moreover, data generated with HBM studies should be communicated to health professionals, regulators and policymakers, as they are of great relevance to health risk management, in particular through the implementation of measures to prevent exposure and to mitigate the identified risks [7]. HBM has been seldom performed simultaneously with the collection of environmental exposure data [8,9,10]. Additionally, the majority of HBM studies only consider exposure to one or a few chemicals at a time [11]. Still, the HBM4EU initiative, a European project with 30 participating countries, including Portugal, and with the support of the European Environment Agency (https://www.hbm4eu.eu/; accessed on 28 October 2021), is coordinating and advancing HBM across Europe. It has defined a list of priority hazardous substances including, but not limited to, emerging substances, flame retardants, phthalates, polycyclic aromatic hydrocarbons (PAHs), pesticides, benzophenones, mycotoxins, and some heavy metals and metalloids [12]. Several HBM studies have been performed among the Portuguese population; however, the available information remains disperse and limited to some pollutants. Among the selected priority pollutants, PAHs, pesticides, and metals are among the most characterized compounds within the Portuguese population. To the best knowledge of these authors, existing information has never been gathered in a way that would allow a global evaluation of the HBM studies performed among the Portuguese population. Thus, the present work aims to bring together the information retrieved from HBM studies related to the Portuguese population’s exposure to PAHs, pesticides, and heavy metals and metalloids over the past 18 years. A critical review of the available information is performed taking into consideration the existent national and international guidelines. Moreover, by integrating the main challenges and lessons learned from Portuguese HBM studies, the main potential health risks are also reviewed, thus contributing to improve and supporting the implementation of safety, health, and environment policies in Europe.

## 2. Methodology

The available scientific literature was searched on Thomson Reuters ISI Web of Knowledge, PubMed, Science Direct, and Google Scholar databases. Combinations of at least two of the following keywords were used: “Portugal”, “Portuguese”, “human biomonitoring”, “biomarkers of exposure”, “polycyclic aromatic hydrocarbons”, “PAH”, “pesticides”, “heavy metals”, “cadmium”, “chromium”, “arsenic”, and “lead”. All the HBM studies assessing exposures to PAHs, pesticides, heavy metals and metalloids within the Portuguese population were selected.

The inclusion criteria for the selected studies were the determination of at least one of the selected pollutants and/or its biomarkers of exposure in biological fluids and to have full access to the study; studies not reporting original data or surveyed in populations not including Portuguese subjects were excluded. Overall, the literature search identified a total of 25 HBM studies published between 2003 and 2021 and assessing the Portuguese population exposure to PAHs (10 studies; 40%), heavy metals and metalloids (10 studies; 40%), and pesticides (5 studies; 20%).

## 3. Selected Chemicals

### 3.1. Polycyclic Aromatic Hydrocarbons

PAHs are organic pollutants released from petrogenic sources and by reactions of incomplete combustion of organic materials and pyrolysis. The production of energy from petroleum and its derivatives (e.g., fossil fuels), coal tar, and wood, as well as emissions from the commercial, institutional, and household sector, agricultural activities, and from road transports constitute the major sources of ambient PAHs [13,14,15]. Tobacco smoke, open fires, and burning of combustion materials (e.g., gas, wood, coal, etc.) used for cooking and heating, as well as penetration from outdoor emissions, are the predominant sources of PAHs in indoor environments [13,16,17,18]. PAHs are listed in the international priority pollutant lists [19,20] and are already among the selected pollutants included by WHO in the guidelines to promote indoor air quality [21]. Among PAHs, benzo(a)pyrene is the only compound with proved carcinogenicity in humans (Group 1–International Agency for Research on Cancer (IARC)) [22]. Naphthalene, benz(a)anthracene, benzo(b)fluoranthene, benzo(j)fluoranthene, benzo(k)fluoranthene, chrysene, dibenz(a,h)anthracene, dibenzo(a,l)pyrene, and indeno(1,2,3-c,d)pyrene are classified as probable/possible carcinogens (Group 2A/B) [22,23]. People are exposed to PAHs through inhalation, ingestion, and/or dermal contact. Therefore, the determination of total exposure to these ubiquitous pollutants is only possible through biomonitoring studies. After absorption, PAHs are distributed by blood route and are prone to accumulate in the fat tissues [14]. PAHs metabolization occurs through complex biochemical reactions in the liver and in a lesser extent in the lungs, intestinal mucosa, skin, and kidneys in order to expedite their elimination from the human body [14]. PAHs are excreted through the urine, bile, milk, and feces in the form of hydroxylated compounds conjugated with macromolecules (glutathione, glucuronide, or sulphate) or as unmetabolized compounds [24,25,26,27,28,29].

Regarding the Portuguese population, there are 10 HBM studies assessing the environmental and/or occupational exposure to PAHs. Recently, some authors [28] assessed the levels of eighteen PAHs and six major metabolites in the breast milk of nursing mothers. Levels of total unmetabolized and metabolized PAHs varied between 55.2 and 1119 ng/g fat and from 6.66 to 455 ng/g fat, respectively. Naphthalene, dibenz(a,h)anthracene, benzo(g,h,i)perylene, and phenanthrene were the predominant unmetabolized PAHs found in breast milk while 1-hydroxyphenanthrene, 1-hydroxynaphthalene, and 1-hydroxyacenaphthene were the most abundant metabolites [28]. Benzo(a)pyrene and its major metabolite, 3-hydroxybenzo(a)pyrene, were not found in the collected breast milk samples. Moreover, increased levels of PAHs were found in the milk of older nursing mothers (>30 years) and in those whose children were born with less than 3.0 kg of weight [28].

PAH metabolites were also determined in the urine of Portuguese schoolchildren, grill workers, and firefighters (Table 1); no data were reported for other biological matrices or other groups of the population. Overall, median concentrations of total PAH metabolites ranged from 4.02–4.75 µmol/mol creatinine in schoolchildren (3–6 years old) and reached maximum levels of 15.4 µmol/mol creatinine in children attending a preschool situated in Oporto Metropolitan Area (north of Portugal) that is strongly affected by traffic emissions (Table 1). Oliveira et al. [30] determined the levels of six urinary biomarkers of exposure to PAHs in grill workers attending six restaurants from Oporto Metropolitan Area. Daily exposures to grilling emissions strongly impacted total exposure to PAHs, with concentrations of total metabolites being nine times higher during working periods comparatively with resting days (2.77 versus 0.298 µmol/mol creatinine; Table 1). Individual excretion profiles also showed a cumulative increase in the levels of total PAH metabolites during consecutive working days [30]. Regarding firefighting forces, median values of total PAH metabolites varied between 1.59 µmol/mol creatinine in non-smoking and non-occupationally exposed firefighters to 6.96 µmol/mol creatinine in smoking and occupationally exposed individuals who were actively involved in firefighting activities; maximum levels reached 121 µmol/mol creatinine (Table 1). Oliveira et al. [27] reported concentrations of total PAH biomarkers that were up to 340% higher (*p* ≤ 0.05) in subjects participating in firefighting comparatively with non-exposed firefighters. Moreover, those authors also found increased levels of oxidative stress in the blood cells of some exposed firefighters [27]. Available literature demonstrated a positive association between firefighters’ exposure to fire emissions and heat, principally at physically/physiological exhausting conditions with altered values of different biomarkers of inflammation, vascular damage, and tissue injury in biological fluids [31]. Most of the available studies from other countries were performed in Germany (21%), France (17%), Italy (10%), Poland (10%), Spain (7.0%), Belgium (7.0%), and the Czech Republic (7.0%); the remaining studies (one per country; 21% in total) were conducted in Denmark, Finland, Ukraine, the United Kingdom, and Sweden. Overall, median levels of total urinary PAH metabolites found for the Portuguese population (except for firefighters participating in firefighting activities) were lower than the concentrations reported for non-occupationally exposed populations (3.80 × 10^−2^ µmol/mol creatinine in French non-smoking adults [32] to 13.8 µmol/mol creatinine in Polish young children [33]; 0.180 µg L^−1^ in Belgian adolescents [25] to 12.2 µg L^−1^ in German smoking adults [34]) and occupationally exposed groups (0.17 µmol/mol creatinine in French non-smoking metallurgy workers [35] to 28.6 µmol/mol creatinine in German converter workers [36]; 6.41 µg L^−1^ in Slovakian steel workers from a control group [37] to 155.1 µg L^−1^ in Polish coke-oven smoking workers [38]). People who were occupationally exposed to PAHs and/or had regular smoking habits presented higher concentrations of urinary PAH metabolites.

Among the compounds under study within the Portuguese population, 1-hydroxynapthalene and 1-hydroxyacenaphthene were by far the most predominant metabolites, being followed by 2-hydroxyfluorene, 1-hydroxyphenanthrene, and 1-hydroxypyrene (Table 1). Indeed, whenever metabolites of naphthalene (1-hydroxynaphthalene and/or 2-hydroxynaphthalene) were included in the HBM studies, they contributed the most to the levels of total PAH metabolites [9,10,31,33,34,37,38,39,40,41,42,43,44,45,46,47]. As demonstrated by several authors, the highest concentrations of urinary PAH metabolites correspond to the compounds of respective PAHs having lower molecular weights [9,33,34,40,43,44,46]. These findings may be attributed to different half-life times, excretion rates, and different metabolization mechanisms depending on the route of exposure. It has been reported that elimination kinetics of PAH metabolites vary widely between compounds: 3.3–6.6 h for 1-hydroxynaphthalene, 2.3–8.4 h for 2-hydroxyfluorene, 4.3–13.8 h for 1-hydroxyphenanthrene, 3–35 h for 1-hydroxypyrene, and 3–24 h for 3-hydroxybenzo(a)pyrene [48,49,50,51,52,53]. Moreover, lighter PAHs (2–3 aromatic rings) are known to be preferentially eliminated through the urine while compounds with higher molecular weights (5–6 rings) are predominantly excreted through the feces [52,54].

Median concentrations of 1-hydroxypyrene, (considered the biomarker of exposure to PAHs) among the Portuguese preschool children varied between 5.72 × 10^−2^ to 0.184 µmol/mol creatinine and reached maximum concentrations of 0.941 µmol/mol creatinine in children attending a preschool situated directly next to a mall and a gas station and in a major entrance road of a city in the north of the country (Table 1). The range of 1-hydroxypyrene concentrations determined in the Portuguese population was close to the values reported for other non-occupationally exposed European citizens, except for preschool Ukrainian children (0.31–0.74 µmol/mol creatinine). Mucha et al. [55] reported higher concentrations of 1-hydroxypyrene in children living at Mariupol, one of the most polluted cities of Ukraine, and in close proximity to two major steel plants and an associated coking facility. Measurement of urinary 1-hydroxypyrene has been used to monitor occupational exposure in firefighters and in coke-oven, aluminum production, and metallurgy workers. Overall, median concentrations of 1-hydroxypyrene in Portuguese grill workers varied from 0.049–0.086 µmol/mol creatinine (maximum up to 1.09 µmol/mol creatinine) while for firefighters, levels ranged from 0.02–0.04 µmol/mol creatinine and reached maximum concentrations of 0.85 µmol/mol creatinine in smoking firefighters participating in fire suppression (Table 1). Grill workers working at restaurants are routinely exposed directly to emissions of grilling during lunch and dinner times across a regular working week while firefighters’ exposure to fire emissions is dependent on seasonal forest fires. Regarding other European occupational exposed groups, concentrations of 1-hydroxypyrene varied between 0.093 µmol/mol creatinine in a reference group of workers and 7.00 µmol/mol creatinine in German converter workers (maximum levels of 16.3 µmol/mol creatinine) [36]; and from 0.586 µg L^−1^ in Slovakian steel workers [37] to 15.4 µg L^−1^ in Polish smoking coke-oven workers [38,41]. A maximum level of 82.0 µg L^−1^ was reported. Available data reveal the strong relation between daily exposure to PAHs during regular work shift and increased urinary concentrations of 1-hydroxypyrene, when compared with control subjects [35,36,39,47,56,57,58]. Despite being the most characterized biomarker, several authors indicated that 1-hydroxypyrene contributed less to the levels of total PAH metabolites, being also the metabolite that presented one of the lowest percentage increases when environmental or occupational exposure was considered [34,43,47,59]. Therefore, the available information raises some doubts regarding the adequacy of using solely 1-hydroxypyrene as the biomarker of exposure to PAHs. Although being widely used to evaluate environmental and/or occupational exposures, no reference standard guidelines or recommended maximum limits are established for 1-hydroxypyrene or for any other PAH metabolite. Jongeneelen [60] proposed a benchmark limit of 1-hydroxypyrene (0.24 μmol/mol creatinine) for non-smoking and non-occupationally exposed people. The Biological Exposure Index Committee of the American Conference of Governmental Industrial Hygienists proposed a benchmark of 0.5 µmol/mol creatinine (1.0 µg L^−1^) of 1-hydroxypyrene as indicative of occupational exposure to PAHs [61]. Regarding the European population, median concentrations of urinary 1-hydroxypyrene exceeded the limit of 0.5 µmol/mol creatinine in Ukrainian preschool children living near a steel mill and a coking facility: 0.62 and 0.74 µmol/mol creatinine were reported for boys and girls, respectively [55]. This proposed limit was also exceeded in some German smoking bitumen workers (0.58 µmol/mol creatinine [57]), Italian and Polish coke-oven workers (0.75–1.02 µmol/mol creatinine and 15.4 µg L^−1^ [38,41,56]), and in German converter, carbon electrodes, refractory materials, and coke production workers (1.97–7.00 µmol/mol creatinine [36]). More recently, Jongeneelen [62] proposed the value of 1.4 µmol/mol creatinine of 1-hydroxypyrene as the non-biological effect level for occupationally exposed workers. Among the limited data available, only some German workers from different industries presented median urinary levels that exceeded up to five times the proposed non-biological effect protective value [36].

The metabolite 3-hydroxybenzo(a)pyrene, a PAH biomarker of carcinogenicity, was only found in the urine of some grill workers (ca. 13% of study population; median of 1.71 nmol/mol creatinine and range of 0.98–2.67 nmol/mol creatinine [30]). This biomarker was never found in the urine of children and firefighters, making these findings in line with those of other studies [37,38,41,42,44,63]. The low detection rates of the metabolites of high molecular weight PAHs (including 3-, 7-, 9-hydroxybenzo(a)pyrene and 1-, 2-, 3-, 4-, 5-, 6-hydroxychrysene) can be explained by their higher elimination rates through the feces rather than in the urine [52,54]. Moreover, some authors reported that urinary excretion of 3-hydroxybenzo(a)pyrene in animals only represented 0.1–0.2% of the benzo(a)pyrene dose given to the animal, thus reflecting the complex metabolism of this metabolite and its higher excretion rate through the feces [54]. Among the European population, 3-hydroxybenzo(a)pyrene was only detected in the urine of some French citizens (9.0 × 10^−6^ to 1.1 × 10^−3^ µmol/mol creatinine for non-smokers versus 2.3 × 10^−5^ to 2.3 × 10^−2^ µmol/mol creatinine for smokers) and Italian citizens (3.54 × 10^−5^ versus 3.37 × 10^−5^ µmol/mol creatinine for non-smokers and smokers, respectively). Barbeau et al. [35] performed HBM studies on French metallurgic workers and reported urinary 3-hydroxybenzo(a)pyrene levels that varied from 0.02 × 10^−3^ to 0.74 × 10^−3^ µmol/mol creatinine. Authors reported that workers in anode production presented a greater exposure to 3-hydroxybenzo(a)pyrene and 1-hydroxypyrene than other metallurgic workers. More HBM studies are necessary to better evaluate occupational exposure to PAHs and the associated health risks, which will contribute to the establishment of reference values.

An alternative to assess environmental and/or occupational exposure to PAHs is through the assessment of unmetabolized compounds in biological samples. So far there are only seven studies regarding the urinary levels of PAHs among the European population: three were conducted in Poland, another three in Italy, and one in Belgium. Overall, urinary levels of total PAHs among the general population ranged from 38.6 ng L^−1^ in non-smoking Italian adults [24] to 98.8 ng L^−1^ in Belgian adolescents [25]. Occupationally exposed groups presented median urinary levels of total PAHs varying from 33.5 ng L^−1^ in a Polish control group of coke-oven workers [64] to 1998 ng L^−1^ in smoking coke-oven workers [38,41]. As observed in the metabolite profiles, PAHs with low molecular weight (2–3 rings: naphthalene, fluorene, phenanthrene) were the most predominant compared with the heavier compounds (fluoranthene, pyrene, benz(a)anthracene, chrysene); urinary benzo(b)fluoranthene, benzo(k)fluoranthene, benzo(a)pyrene, and dibenz(ah)anthracene are frequently not detected in urine samples. Pyrene was found in median concentrations that varied from 0.57 ng L^−1^ in Belgian adolescents [25] to 1.6 ng L^−1^ in Italian adults exposed to solid waste incinerator emissions (maximum values of 2.8 ng L^−1^) [65]. Regarding occupational exposed groups, concentrations of urinary pyrene ranged between 1.9 ng L^−1^ in Polish coke-oven workers [64] to 54 ng L^−1^ in the end-shift urine of Italian road paving workers [66] and reached maximum levels of 328 ng L^−1^ in Polish smoking coke-oven workers [38,41]. Benzo(a)pyrene was detected in the urine of Belgian adolescents (0.21 ng L^−1^) [25] and in Polish coke-oven workers (<0.5–91.7 ng L^−1^ [38,41,64]). Urinary concentrations of benzo(a)pyrene and other possible/probable carcinogens (naphthalene, benzo(a)anthracene, chrysene, benzo(b)fluoranthene, benzo(j)fluoranthene, benzo(k)fluoranthene, dibenz(a,h)anthracene, dibenzo(a,l)pyrene, and indeno(1,2,3-c,d)pyrene) ranged from 5.1 ng L^−1^ (Polish coke-oven control subjects [64]) to 895 ng L^−1^ (smoking Polish coke-oven workers [38]). Altogether these compounds accounted for 11–81% of urinary concentrations of total unmetabolized PAHs in occupationally exposed groups. Similar findings were reported in the urine of Belgian adolescents (79.8 ng L^−1^, 81% of total PAHs) [25].

Exposure to PAHs has been directly associated with many potential health risks [67]. Due to their physical–chemical properties, PAHs may cause developmental, immunological, and reproductive effects in humans, principally in the most vulnerable groups of the population [68]. In 2013, the World Health Organization included some PAHs in the list of endocrine-disrupting substances [69]. Ambient air levels of particulate bound benzo(a)pyrene and maximum concentrations of benzo(a)pyrene, benz(a)anthracene, benzo(b)fluoranthene, and chrysene in different foodstuffs are legislated in the European Union [70,71]. Environmental and principally occupational exposure to PAHs have been related to the increase of morbidity and mortality rates [14,15,67]. Indeed, exposure to PAHs induces human carcinogenesis through the formation of active carcinogenic intermediary molecules that promote the formation of DNA adducts, thus resulting in DNA mutations, alteration of gene expression profiles, and tumorigenesis [72,73,74]. PAHs are also responsible for the initiation of cardio-respiratory inflammatory processes [67,75,76,77]. Exposure to naphthalene, a possible human carcinogen [23] strongly contributes to increased human cancer risk [21,78]. Since unmetabolized naphthalene and its major metabolites (1- and 2-hydroxynaphtalene) constitute one of the most abundant PAHs in the urine (Table 1), the inclusion of this compound in HBM studies is strongly recommended. Future HBM studies should include the determination of unmetabolized benzo(a)pyrene and other possible/probable carcinogens and the metabolized compounds of the most predominant PAHs (naphthalene, fluorene, phenanthrene, and pyrene) to better estimate the total exposure and the potential health risks. Moreover, there is a clear need to define maximum exposure limits for unmetabolized and metabolized PAHs in biological matrices, and particularly in the less invasive ones, principally for the most susceptible groups of the population, such as children, pregnant women, and people with chronic diseases, as well as for groups that are occupationally exposed to PAHs.

**Table 1:** Urinary concentrations of PAH metabolites (OHPAHs; median and/or range, expressed as µmol/mol creatinine) reported in the Portuguese population.

			PAH Metabolites ^1^					
City	Population *n* (Age, Years)	Notes	1OHNaph + 1OHAce ^a^	2OHFlu	1OHPhe	1OHPy	∑OHPAHs	Reference
Chaves	Children (3–6)	Morning void	n.r.	(0.12–12.0)	(0.08–0.59)	(0.08–0.91)	(0.28–13.5) *	[79]
		Last-night void	n.r.	(0.01–0.61)	(0.03–0.27)	(0.01–0.35)	(0.05–1.23) *	
	Children	Morning and last-night voids						[9]
Porto	27 (3–5)	Boys (*n* = 17)	4.42 (5.11 × 10^−2^–14.4)	0.126 (1.26 × 10^−2^–1.34)	5.53 × 10^−2^ (1.75 × 10^−2^–0.301)	5.72 × 10^−2^ (1.93 × 10^−2^–0.246)	4.75 (0.240–15.4)	
		Girls (*n* = 10)	3.90 (0.178–7.46)	0.124 (5.82 × 10^−2^–0.866)	5.63 × 10^−2^ (3.80 × 10^−2^–0.121)	6.58 × 10^−2^ (2.05 × 10^−2^–0.128)	4.15 (0.345–7.71)	
Chaves	16 (3–5)	Boys (*n* = 5)	3.49 (1.27–7.76)	0.324 (0.104–0.910)	8.53 × 10^−2^ (7.05 × 10^−2^–0.270)	0.117 (4.15 × 10^−2^–0.941)	4.02 (1.54–9.07)	
		Girls (*n* = 11)	3.73 (8.51 × 10^−2^–9.40)	0.221 (0.114–0.482)	0.138 (6.14 × 10^−2^–0.430)	0.184 (4.94 × 10^−2^–0.276)	4.27 (0.556–9.67)	
Bragança	Adults	Control group	1.40 (0.03–4.14)	0.06 (5.67 × 10^−4^–0.48)	0.04 (6.71 × 10^−3^–0.21)	0.03 (1.84 × 10^−3^–0.23)	1.59 (0.10–4.27)	[27]
		Non-smoking exposed firefighters	1.54 (0.60–121)	0.09 (5.67 × 10^−4^–0.47)	0.06 (0.02–0.29)	0.04 (1.84 × 10^−3^–0.19)	1.68 (0.82–121)	
		Smoking exposed firefighters	5.61 (1.18–47.8)	0.62 (0.29–1.61)	0.04 (0.02–0.19)	0.04 (3.69 × 10^−3^–0.85)	6.96 (1.52–48.6)	
Bragança	Adults 75 (22–48)	Post-shift void; Non-smoking and non-exposed to firefighting activities	(0.138–3.59)	(1.39 × 10^−2^–0.182)	(1.21 × 10^−2^–8.38 × 10^−2^)	(1.35 × 10^−2^–0.146)	(0.259–3.71)	[10]
Bragança	Adults 78 (33–41)	Post-shift void						
		Non-smoking and non-exposed to firefighting activities	n.r.	n.r.	n.r.	(1.3 × 10^−2^–6.3 × 10^−2^)	(1.3 × 10^−2^–6.3 × 10^−2^) *	[58]
		Smoking and non-exposed to firefighting activities	n.r.	n.r.	n.r.	(8.0 × 10^−3^–3.9 × 10^−2^)	(8.0 × 10^−3^–3.9 × 10^−2^) *	
Bragança	Adults 153 (21–55)	Post-shift void						[47]
		Non-smoking and non-exposed to firefighting activities	(0.138–1.46)	(2.31 × 10^−2^–0.200)	(1.06 × 10^−2^–7.47 × 10^−2^)	(1.21× 10^−2^–5.44 × 10^−2^)	(0.249–1.57)	
		Non-smoking and exposed to firefighting activities	(0.708–8.25)	(2.65 × 10^−2^–1.33)	(3.30 × 10^−2^–8.21 × 10^−2^)	(1.73 × 10^−2^–0.152)	(0.973–8.75)	
Bragança	Adults 108 (21–60)	Post-shift void						[39]
		Non-smoking and non-exposed to firefighting activities	(0.394–0.971)	(1.75 × 10^−2^–0.201)	(7.95 × 10^−3^–7.40 × 10–^2^)	(8.85 × 10^−3^–6.80 × 10^−2^)	(0.161–0.817)	
		Non-smoking and exposed to firefighting activities	(0.676–2.96)	(2.20 × 10^−2^–0.520)	(1.61 × 10^−2^–0.204)	(2.37 × 10^−2^–0.144)	(0.817–2.06)	
		Smoking and exposed to firefighting activities	(1.61–4.38)	(0.257–1.53)	(3.03 × 10^−2^–0.146)	(4.41 × 10^−2^–0.462)	(1.91–5.71)	
Bragança	Adults 33 (21–40)	Post-shift void						[40]
		Non-smoking and non-exposed to firefighting activities	1.38 (0.58–2.28)	0.11 (1.5 × 10^−3^–0.13)	0.09 (0.02–0.17)	0.04 (0.02–0.10)	1.59 (0.76–2.57)	
		Non-smoking and exposed to firefighting activities	2.75 (0.60–121)	0.06 (8.2 × 10^−4^–0.19)	0.06 (0.03–0.28)	0.02 (1.7 × 10^−3^–0.19)	3.12 (0.86–121)	
Porto	Adults 18 (20–48)	Non-smoking and non-exposed to grilling activities	0.098 (0.029–1.41)	0.018 (1.24 × 10^−4^–0.133)	0.031 (0.016–0.088)	0.049 (0.013–0.188)	0.298 (0.097–1.66)	[30]
		Non-smoking and exposed to grilling activities	2.23 (0.025–42.1)	0.112 (8.49 × 10^−5^ –15.5)	0.073 (2.51 × 10^−4^–0.719)	0.086 (0.011–1.09)	2.77 (0.213–42.3)	

n.r.: not reported. ^1^ 1OHNaph: 1-hydroxynaphthalene; 1OHAce: 1-hydroxyacenaphthene; 2OHFlu: 2-hydroxyfluorene; 1OHPhen: 1-hydroxyphenanthrene; 1OHPy: 1-hydroxypyrene; 3OHB(a)P: 3-hydroxybenzo(a)pyrene; ∑OHPAHs: sum of all PAH metabolites. * Range of ∑OHPAH levels were determined as the sum of the minimum and maximum concentrations reported for each metabolite detected. ^a^ Concentrations of 1OHNaph and 1OHAce were determined together.

### 3.2. Pesticides

Since the 1970s, the use of organochlorine pesticides (OCPs) has been forbidden in industrialized countries, and restricted in several others. However, these compounds endure until today in the environment. In Portugal, the use of several OCPs has been prohibited since 1988; in 2003 the use of lindane in agriculture was also prohibited [80]. Aside from occupationally exposed subjects, exposure to these compounds arises mostly through dietary intake. OCPs are lipophilic compounds that accumulate and remain in adipose tissues over a long period, even decades, and may biomagnify across the food chain, particularly in foods with high lipidic content. They act as endocrine disruptors, induce immune suppression, and are suspected of being carcinogens [81]. Exposure biomonitoring can be performed determining free OCPs and/or their metabolites in biological matrices such as blood, serum, and plasma [80,82]. So far, only three HBM studies have been performed among the Portuguese population related to exposure to OCPs (Table 2). In a HBM study performed between 1997 and 2001 in 160 college students, a total of 12 OCPs were determined in serum samples, with endosulfan sulphate, p,p’-DDE, o,p’-DDT, and p,p’-DDD the compounds most frequently found [82]. Among the OCPs considered, endosulfan sulphate presented the highest average concentrations (42.6 µg L^−1^) with maximum values reaching 1295.5 µg L^−1^ (Table 2). Among DDT isomers and analogues, o,p’-DDT and p,p’-DDT showed maximum levels of 24.8 and 21.9 µg L^−1^, respectively. Average total DDT concentrations were greater than that from total HCH, with the highest concentrations of total DDT observed in the samples collected from females living in urban areas, while higher levels of total HCH were found in males [82]. Cruz et al. [80] evaluated the body burden of the same 12 OCPs in the blood serum of Portuguese residents of an urban community, and in two rural communities located in a region characterized by its strong agricultural activity. The HBM study performed between 2001 and 2002 demonstrated that p,p’DDE, HCH, p,p’DDD, and β-HCH were the prevalent pesticide residues (Table 2). Concentrations of p,p’DDE ranged between undetected to 390.5 µg L^−1^ in urban areas, and from undetected to 43.5 µg L^−1^ and to 171.2 µg L^−1^ in each one of the rural areas (Table 2). The highest Σ-HCH levels were 114.4, 261.8, and 45.5 µg L^−1^ in urban and both rural areas, respectively. Serum concentrations of total DDT were always above the average levels of total HCH. Regarding p’DDE, it was mostly detected in females from all three populations, with levels ranging between <12.5 and 390.5 µg L^−1^ (Table 2). The comparison of distinct ages demonstrated that the youngest subjects aged between 20 and 39 years old were also exposed to OCPs [80].

Prenatal exposure to OCPs has been related to undesirable developmental defects, such as reduced birth weight, preterm birth, growth retardation, changed psychomotor and cognitive functions, and alterations of the thyroid hormonal status [83]. One HBM study assessed the levels of pp’DDE in the maternal and umbilical cord serum of 68 women/newborn sets inhabiting the south Portuguese region of Algarve [83]. Overall, mean total pp’DDE levels were 1.11 ± 0.69 µg L^−1^ and 0.85 ± 0.50 µg L^−1^ for maternal and cord serum, respectively, with significant correlations being observed when compared (*p* < 0.05) (Table 2). A multivariate analysis showed that higher serum concentrations of pp’DDE were associated with the oldest primiparous women living in rural areas and a greater consumption of vegetables and fruits [83]. Therefore, it was proven that selected OCPs are able to go through the placenta barrier and reach the fetus [83].

Several European HBM programs and regional studies have shown that OCPs, namely DDE and HCB, are found in higher values in older people comparatively with other age ranges [84]. In Italy, 8 OCPs were evaluated in 137 blood serum samples obtained from general inhabitants of Novafeltria, Pavia, and Milan [85]. Results showed that the most prevalent pesticides and the main contributors to total OCP levels were p,p’-DDE and HCB; significant differences were observed among the three considered towns (Milan > Novafeltria > Pavia). As in the Portuguese studies, females presented significantly higher levels of HCB and p,p′-DDE when compared to males. Positive correlations were found between p,p′-DDE and HCB and the age of Novafeltria individuals [85]. In central Greece (Larissa), OCPs were also determined in serum samples from 103 volunteers, with p,p′–DDE (incidence of 99%, median of 1.25 µg L^−1^) and HCB (incidence of 69%, median of 0.13 µg L^−1^) the most prevalent pesticides; significant associations were found between p,p′–DDE and HCB concentrations and the age of the participants [86]. A HBM study performed among the Spanish population between 1992 and 1996 assessed the serum levels of p,p′-DDT, p,p′-DDE, β-HCH, and HCB in 953 healthy adults [87]). Overall, the pesticide p,p′-DDE was found in 98% of the subjects, followed by HCB (89%) and β-HCH (77%); p,p’-DDT was detected in 26% of the samples. The pesticides p,p′-DDE, β-HCH, and HCB presented a geometric mean of serum concentrations of 822, 167, and 379 ng g^−1^ lipid, respectively [87]. Each OCP was positively correlated with the age and body mass index of participants, and negatively associated with the period of blood collection [87]. No correlation was observed between OCP concentrations and dietary habits [87]. The distribution of serum OCPs (HCB, DDE, DDT, α-HCH, β-HCB, and γ-HCH) was also evaluated from 2006–2007 in samples from 386 persons from the French adult population [88]. Median serum levels were 22.8 ng g^−1^ lipid for HCB, 0.74 and 27.0 ng g^−1^ lipid, respectively, for α- and β -HCH, and 3.8 and 104.6 ng g^−1^ lipid, respectively, for DDT and DDE. Lindane (γ-HCH) was found in roughly 10% of the subjects [88].

Glyphosate is a broad-spectrum non-selective herbicide with increasing use, being nowadays one of the most commonly used herbicides at a global scale, as recently reviewed [89]. The broad range of glyphosate salts is the reason why the metabolism of glyphosate is not fully known [90]. However, the two major metabolites formed are known: α-amino-3-hydroxy-5-methyl-4-isoxazolpropionic acid (AMPA), and glyoxylate [91]. According to data retrieved from animal models, the absorption rate of glyphosate is estimated as 20% [92]. In humans, once absorbed, it is promptly excreted unmetabolized through urine [93,94]. The European Food Safety Authority (EFSA) considers that the existing scientific evidence is inadequate to consider the herbicide as possibly carcinogenic to humans [95], despite the classification of glyphosate as a group 2A chemical (probably carcinogenic to humans) [96] by the International Agency for Research on Cancer (IARC). Furthermore, neurological effects [97] and disruption of the endocrine system [98] were reported following exposure to glyphosate-based formulations. Some studies call for an update of the existing safety standards for glyphosate-based formulations [99].

In Portugal, there is a lack of biomonitoring studies confirming exposure to glyphosate, with only two single studies published in 2020 [100] and 2021 [89].

In an adult biomonitoring pilot-study [100], 79 Portuguese citizens were analyzed for glyphosate and AMPA. The participants, aged between 47 and 50 years old, were enrolled in two rounds. In the first round, glyphosate was found in the urine of 28% of the participants (at an average level of 0.25 µg L^−1^) and AMPA in 50% (at an average level of 0.16 µg L^−1^). In the second round, glyphosate was determined in 73% of the participants (at an average level of 0.13 µg L^−1^) and AMPA in 97% (at an average level of 0.10 µg L^−1^). The frequency of contamination was comparable to that found in similar studies carried out in adult populations in Germany [101] and Ireland [102], although the mean glyphosate levels were markedly higher in the Irish study (0.87 µg L^−1^) [102].

The exposure of Portuguese children to glyphosate is of particular concern, due to their higher susceptibility owing to physiological immaturity and higher consumption per kilogram of body weight [103,104]. In Portugal, a single biomonitoring study was carried out in children. The study enrolled 41 children, aged between 2 and 12 years old, living in different areas of the Portuguese mainland. Glyphosate was found in 95.1% (*n* = 39) of the urine samples analyzed at an average level of 1.77 ± 0.86 µg/L. The number of positive samples was comparable to the results of the scarce previous studies carried out in other countries. Nevertheless, it is noteworthy that the maximum value of glyphosate determined in the urine of Portuguese children (4.35 µg L^−1^) was higher than the values previously reported, such as in Denmark (3.31 µg L^−1^) [105], Mexico (2.63 µg L^−1^) [106], and the USA (2.13 µg L^−1^) [104]. Regarding the exposure determinants, higher glyphosate levels were found in girls, in older children as well as the ones living near (up to 1 km) of agricultural fields and consuming higher amounts of home-produced foods. Lower concentrations were determined in children from parents with increased educational level. In the risk assessment, the authors estimated that the lower-bound urinary glyphosate levels represented at least 1–2% of the acceptable daily intake, established transversely for all the population, regardless of the higher susceptibility of children [89].

### 3.3. Metals and Metalloids

**Figure 1:**
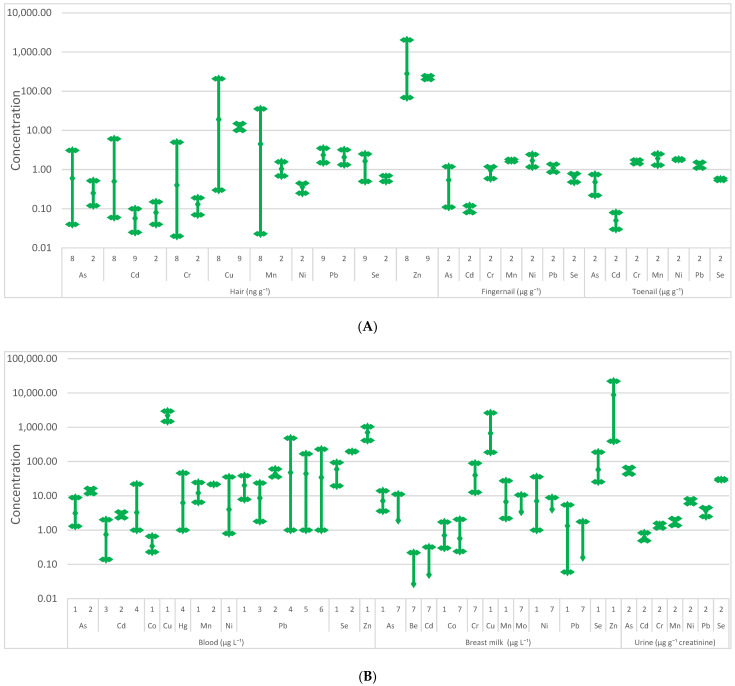
Minimum, maximum, and average concentration of heavy metals and metalloids in solid (**A**) and liquid (**B**) biological matrices among the Portuguese population. 1 [119]; 2 [114]; 3 [113]; 4 [117]; 5 [116]; 6 [115]; 7 [120]; 8 [111]; 9 [118].

Heavy metals and metalloids can originate from natural and anthropogenic sources, such as volcanic activities, industrial activities, road traffic, the use of fertilizers and pesticides, among others [107,108]. Some of these elements are essential for humans in small concentrations; however, many of them have toxicological potential and their ubiquitous presence in soils, aquatic environment, food and air enhances human exposure to these pollutants [108,109]. Heavy metals such as cadmium (Cd), chromium (Cr), and nickel (Ni) and some metalloids (e.g., As) alter cell structure and replace cofactors for enzymatic activities, and being chemically reactive and difficult to remove from the organism, are thus associated with carcinogenicity [83,109]. Although the adverse health effects of exposure to heavy metals and metalloids are not new, this exposure has been increasing in some regions, namely in developing countries. Some examples are the use of mercury (Hg) in gold mining and As/Cr in wood treatments and lead (Pb) in petrol [110]. Several HBM studies assessed the levels of heavy metals and metalloids among the Portuguese population, namely, As, beryllium (Be), Cd, Cr, cobalt (Co), copper (Cu), Pb, manganese (Mn), Hg, Ni, selenium (Se), and zinc (Zn). Most of these studies were focused on populations living near mines [111,112,113,114], incinerators [115,116,117], and volcanic areas [118]; another two works studied lactating women [119,120]. The obtained data are presented in Figure 1.

The HBM data available for human solid matrices showed that fingernails and toenails presented the highest average concentrations, namely for Mn (1.93 µg g^−1^) and Ni (1.82 µg g^−1^), while the lowest average concentration was found for Cd (0.05 µg g^−1^). The occurrence of trace elements in the hair of the Portuguese population was lower than the values found in nails, with the highest average concentration found for Zn (281.9 ng g^−1^) (Figure 1A). Regarding human liquid matrices, blood presented slightly higher concentrations than breast milk, with the highest averages and maximum levels reported for Zn (708 and 1038 µg L^−1^ for blood; 8784 and 22,050 µg L^−1^ for breast milk, respectively) and Cu (666 and 2628 for blood; 666 and 2628 µg L^−1^ for breast milk, respectively); the lowest average values were described for Be (0.027 µg L^−1^) in breast milk and for Co (0.34 µg L^−1^) in blood (Figure 1B). Comparing the reported blood concentrations among the environmentally exposed and control Portuguese population cohorts with the available reference values for Cd (1.0 µg L^−1^), Pb (90 µg L^−1^) and Hg (2.0 µg L^−1^) [121], it was found that the majority of the HBM studies presented levels that surpassed the limits [112,113,115,116,117]. In urine, the highest averages and maximum concentrations were found for As (54.1 and 67.5 µg g^−1^ creatinine, respectively) and Se (29.6 and 31.2 µg g^−1^ creatinine, respectively) while the lowest levels were reported for Cd (0.6 µg g^−1^ creatinine) (Figure 1B).

Portuguese history is linked to mining activities, which have positively affected the regional and national economies; however, they promote environmental contamination, even after mines closure [113,114]. This environmental contamination affects humans through the inhalation of airborne dust and the ingestion of contaminated water and foods (e.g., local vegetables and animals) [114]. So far, only four studies have assessed the occurrence of nine trace elements (As, Cd, Cr, Cu, Mn, Ni, Pb, Se, and Zn) in the blood, fingernails, hair, toenails, and urine of populations living near Portuguese mines [111,112,113,114]. The obtained results showed that these populations were exposed to higher concentrations of As, Cd, Cu, Mn, Pb, and Se than subjects from control groups [111,112,113,114]. Another study was able to demonstrate the occupational and environmental exposure of populations living near and working in the Panasqueira mine, which presented higher exposure, namely to As [114]. The reported HBM studies (assessing both environmental and occupational exposure) suggested higher exposures among the Portuguese population comparatively with other international studies performed in Canada, Germany, and Saudi Arabia, namely for Cd and Pb in blood [121,122] and for Cd, Cr, and Pb in urine [123]. The concentrations found can have an impact on community health with some correlations with the prevalence of pathologies such as eye irritation, and mucous and respiratory symptoms, mainly in vulnerable population groups such as older people and children. However, chronic effects, which may go unnoticed, represent a crucial point that should also be assessed [113,114].

Incinerators can potentially release heavy metals and metalloids into the environment; their influence on the populations residing in the vicinity has been raising public and scientific concern [117,124,125]. There are works that assessed the concentrations of heavy metals in blood (Cd, Hg, and Pb in general blood; Pb in maternal, children, and umbilical cord blood) in populations living near incinerators situated in Lisbon and on Madeira island (Portugal) [115,116,117]. Overall, no significant differences were observed between Cd, Hg, and Pb in the general blood of exposed and control groups. Although there were no differences between the groups, the average values for Cd (6 µg L^−1^) and Hg (11 µg L^−1^) were higher than the available reference values (1.0 and 2.0 µg L^−1^, for Cd and Hg respectively). Additionally, blood concentrations of Cd, Hg, and Pb among the Portuguese population were higher than those reported by HBM studies performed in Germany, Saudi Arabia, and Canada [121,122,123]. Lisbon population presented higher exposure to heavy metals, probably due to additional sources of pollution (e.g., traffic intensity and industrial density), which are less intensive in Madeira [117].

Regarding the studies performed with maternal and cord blood, reduced concentrations of Pb have been described over time, probably due to the use of unleaded gasoline, however some detected levels (1–229 μg L^−1^) were higher than the limits established by the Center for Disease Control and Prevention (100 μg L^−1^) [115]. In children’s blood, higher Pb values were found for those living near the incinerators [115]. About 2.8% of the children participating in the HBM study presented blood Pb levels equal to or higher than 100 μg L^−1^. These values concur with the concentrations obtained in general blood and are also much higher than the levels observed in populations from Canada, the Czech Republic, and Germany, in which 95% confidence intervals were below 45 μg L^−1^ [115,122].

Volcanic emissions may represent a significant risk to human health due to the exposure to trace elements, namely through contaminated soils [126]. A HBM study was conducted on the Portuguese islands of São Miguel and Santa Maria (Açores archipelago) for the determination of Cd, Cu, Pb, Se, Zn in the hair of subjects exposed to volcanic emissions [118]. Hair concentrations of Cd, Cu, Pb, and Zn were increased in the exposed group, with the highest levels found in the hair of the oldest population. Overall, hair levels of Cd and Pb were lower than the concentrations reported in the surrounding area of São Domingos mine in Portugal and for populations from Indonesia, Pakistan, and India [111]. Nonetheless, the concentrations of Cd and Pb in the exposed group were higher than those of groups from developed countries, such as Germany, Italy, and Canada [118].

Trace elements can be transferred from both the body reserves and the blood into the breast milk of a lactating mother [119,120]. Two HBM studies on Portuguese lactating women were performed regarding the presence of As, Cd, Co, Cr, Cu, Mn, Mo, Ni, Pb, Se, and Zn in blood, colostrum, and breast milk samples [119,120]. The obtained data were in close agreement with the ranges of values found in the international literature, with colostrum presenting higher concentrations for most of the elements than breast milk; no correlation was found with the levels found in blood (Figure 1B) [119,120]. Nevertheless, a great variability among the individual excretion rates of heavy metals via human milk was detected [120].

## 4. Final Remarks

This review collects, for the first time, the existing information related to the contribution of HBM to the characterization of the (environmental and occupational) exposure to PAHs, metals and metalloids, and pesticides in the Portuguese population. Regarding exposure to PAHs, it was found that the main metabolites of low molecular weight compounds were the most abundant. Urinary 1-hydroxypyrene, the PAH biomarker of exposure to PAHs, was found at levels that were predominantly lower than the proposed benchmarks for non-smoking and non-occupationally exposed individuals (0.24 μmol mol^−1^ creatinine; [60]) and for occupationally exposed workers [61]. Urinary 3-hydroxybenzo(a)pyrene, the biomarker of exposure to carcinogenic benzo(a)pyrene, was only detected in some grill workers daily exposed to grilling emissions. Regarding concerns about Portuguese exposure to metals and metalloids, increased levels of several elements (with high relevance for Cd and Pb) were mainly found in subjects living in the proximity of mines and volcanoes, but also in children near incinerators. Moreover, exposure to pesticides was also observed, including in the youngest populations; however, the data are still very scarce. It is important to recognize as a significant limitation that determination of the selected pollutants is only a proxy measure in specific biological fluids and may not reflect the real levels in targeted tissues and organs.

Despite the Portuguese participation in the European Human Biomonitoring Program-HBM4EU, the present study revealed the existence of limited information regarding the evaluation of Portuguese exposure to the selected hazardous substances and the lack of standardization in the different methodologies applied. The difficulty in mobilizing a representative sample (namely by gender, age, region, informed agreement) to study a wide range of health indicators and obtain more robust results can be also identified as a limitation. This not only hindered an integrated view of the problem but also hampered consistent comparison between the obtained results, ultimately resulting in difficulty in implementing policies based on scientific evidence. Additionally, available information is insufficient to explore temporal and geographical trends across the Portuguese and European populations. Therefore, more HBM studies are needed to better characterize Portuguese exposure to the selected health-hazardous contaminants/pollutants and compare it with total exposures determined in other European populations. To overcome this gap of knowledge, a regular HBM surveillance program should be performed among different age groups of the European population, which would allow a more comprehensive temporal and geographical evaluation of environmental and occupational exposure to the selected health-relevant pollutants. Other health-relevant pollutants (e.g., flame retardants, phthalates, benzophenones, and mycotoxins) should be included in future HBM studies to perform a more complete exposure assessment that would support a more realistic health risk assessment (including the contribution from potential synergistic effects) of the population. The paucity of specific and properly validated biomarkers, as well as the lack of information on the toxicokinetics that persist for many chemicals, hinders objective risk assessments. In addition, for many chemicals, the lifetime health impacts associated with exposure remain unknown and guidance is largely missing. These limitations were in line with the main hurdles and challenges of HBM considering risk assessment of chemicals identified by EU and extra-EU regulators [3]. In spite of the recognized limitations, HBM makes it possible to assess trends in temporal exposure, to characterize geographical patterns of exposure, compare different population groups, and identify vulnerable subpopulations [7] to serve as the starting point for the implementation of preventive measures and assess the effectiveness of policy actions [127].

## Figures and Tables

**Table 2:** Concentrations (µg L^−1^) of pesticides reported in the Portuguese population.

Pesticide	Matrix	Sample	Incidence (%)	Range	Average ± SD	Reference
Σ-Hexachlorocyclohexane (HCH) isomers (α, β, ϒ)	Serum	Coimbra (*n* = 44; urban) Verride (*n* = 70; rural) Ereira (*n* = 89; rural)	20.45 22.86 10.11	1.08–114.4 1.08–265.8 1.08–45.5	10 ± 22.8 13 ± 36.6 6.1 ± 8.6	[80]
Σ-DDT			34.09 20 37.08	<12.5–814.9 <12.5–70.7 <12.5–427.9	93.5 ± 140.9 43.9 ± 9.7 56 ± 50	
p,p′ DDT			4.55 NQ NQ	<37.5–814.9 NQ NQ	37.5 ± 120 18.8 18.8	
o,p′-DDT			9.09 1.43 4.49	<15–141.0 <15–20.7 <15–256.7	15.4 ± 26.6 7.7 ± 1.6 10.8 ± 26.5	
p,p′-DDE			20.45 18.57 30.34	<12.5–390.5 <12.5–43.5 <12.5–171.2	28.6 ± 75 9.5 ± 8.1 14.6 ± 20.6	
p,p′-DDD			6.82 2.86 8.89	<15–95.3 <15–25.9 <15–199.1	12 ± 17.4 7.95 ± 2.7 11.9 ± 23.2	
Aldrin			6.82 NQ NQ	<5–372.9 NQ NQ	17.4 ± 65.3 2.5 ± 0.0 2.5 ± 0.0	
Dieldrin			6.82 NQ NQ	<14.5–356.4 NQNQ	22.3 ± 61.9 7.3 ± 0.0 7.3 ± 0.0	
Heptachlor epoxide (HE)			11.36 NQ NQ	<12.5–239.1 NQ NQ	14.8 ± 36.6 6.3 ± 0.0 6.3 ± 0.0	
Hexachlorobenzene (HCB)			6.82 NQ NQ	<12.5–393.3 NQ NQ	20 ± 64.3 6.3 ± 0.0 6.5 ± 2.3	
Endosulfan sulphate			2.27 NQ 1.12	<15–547.6 NQ <15–108.8	19.8 ± 81.4 7.5 ± 0.0 8.6 ± 10.7	
Σ-HCH	Serum	160 students	21.3	<1.08–472.2	24.9 ± 71.6	[82]
Σ-DDT			56.3	<12.5–569	74.7 ± 92.2	
p,p′-DDE			30	<12.5–175	18.3 ± 27.8	
p,p′-DDD			25	<15–237	18.0 ± 30.5	
o,p′-DDT			28.1	<15–361	24.8 ± 48.0	
p,p′-DDT			8.1	<37.5–98.5	21.9 ± 12.3	
HCB			10	<12.5–141	10.7 ± 18.3	
Aldrin			16.3	<5–400	13.1 ± 42.2	
Dieldrin			16.9	<14.5–270	14.0 ± 30.8	
HE			10.6	<12.5–309	11.1 ± 30.2	
Endosulfan sulphate			37.5	<15–1295.5	42.6 ± 126.9	
p,p′-DDE	Maternal serum Umbilical cord serum	*n* = 68 *n* = 68	100 100	0.32–2.68 0.22–2.05	1.11 ± 0.69 0.85 ± 0.50	[83]
Glyphosate AMPA Glyphosate AMPA	Urine (1st round) Urine (2nd round)	*n* = 46 adults *n* = 33 adults	28 50 73 97	0.11–1.04 0.10–0.32 0.02–0.63 0.01–0.29	0.25 0.16 0.13 0.10	[100]
Glyphosate	Urine	*n* = 41 children	95.1	0.87–4.35	1.77	[89]

NQ: not quantified.

## Data Availability

Not applicable.
